# A Latent Profile Analysis of Cyber Dating Abuse Among College Students: Associations With Maladaptive Personality Traits, Negative Emotionality, and Impulsivity

**DOI:** 10.1177/08862605251339636

**Published:** 2025-05-28

**Authors:** Ariana Cervantes, Genevieve M. Jessen, Theodore V. Cooper, Mitchell Kirwan

**Affiliations:** 1 The University of Texas at El Paso, USA

**Keywords:** cyber dating abuse, impulsivity, psychopathy, negative emotionality

## Abstract

Cyber dating abuse (CDA) is a novel form of relationship violence enacted via technology, which is largely understudied despite the elevated rates during and after the COVID-19 pandemic. Further, extant research indicates that maladaptive personality traits (psychopathy, narcissism, and Machiavellianism), negative emotionality (depression, anxiety, and stress), and impulsivity are all associated with CDA; yet, how the culmination of these factors may influence one’s propensity for CDA has not been examined. Thus, the present study assessed how maladaptive personality, negative emotionality, and impulsivity may be associated with CDA and whether these characteristics could comprise distinct profiles to predict differences in CDA victimization and perpetration. Predominantly Latinx female college students (*N* = 590, *M*_age_ = 20.35, 75.6% female) completed an online questionnaire assessing CDA, dark triad, depression, anxiety, stress, and impulsiveness. A latent profile analysis generated profiles based on background characteristics (e.g., maladaptive personality) to predict sexual and nonsexual CDA perpetration and victimization. Results from four Kruskal–Wallis tests indicated significant differences between profile membership and sexual and nonsexual CDA perpetration and victimization. Individuals in the profile with relatively low levels of maladaptive personality traits, impulsivity, and subclinical levels of negative emotionality displayed the lowest levels of CDA, which indicates that negative emotionality may function as a catalyst to exacerbate impulsively engaging in CDA. Further, impulsive individuals with greater negative emotionality may also be more likely to seek validation online and to be victimized as a result. Interventions targeting these key variables may be beneficial to reduce rates of both perpetration and victimization.

## Introduction

Cyber dating abuse (CDA) is a novel way of enacting dating violence through technology (e.g., SMS messaging and social media), as a means to control, harass, stalk, and/or abuse one’s partner, which differentiates itself from traditional relationship violence in that psychological harm may be present even in the partner’s physical absence (Zweig et al., 2013). This construct has been primarily investigated among adolescents and young adults, as these age groups are disproportionately impacted by CDA (Li et al., 2023), such that past studies have demonstrated that general dating violence may peak at 20 years of age (Johnson et al., 2015). Further, prevalence rates of CDA vary; among student samples, 12.5% to 26.5% of students report victimization (Víllora et al., 2021) and 15% to 23.8% report perpetration (Peskin et al., 2017; Víllora et al., 2021). Women in particular may be more likely to encounter CDA (Linares et al., 2021) and are more often victimized (29%) when compared to men (23%; Zweig et al., 2013). Similar to relationship violence, CDA often involves reciprocal perpetration, rather than solitary victimization or perpetration (Víllora et al., 2021). Moreover, due to the increase in online dating (Wiederhold, 2021) and the associated rise in the prevalence of CDA during and after the COVID-19 pandemic (Huiskes et al., 2022; Sheridan-Johnson et al., 2024), further investigation regarding predictors of CDA is warranted.

CDA can be further broken down into subtypes (Zweig et al., 2013). For example, sexual CDA pertains to behaviors such as sending unwanted sexually explicit photos or messages without a partner’s consent, requesting unwanted sexual favors, and sharing a partner’s explicit photos without their consent, while nonsexual CDA may include threatening, stalking, or controlling a partner through electronic devices as well as monitoring a partner’s social media accounts (Zweig et al., 2013). These forms of abuse can range from harassment to threatening with the intent to harm and may be co-occurring (Víllora et al., 2021). This is similar to relationship violence, which can also be broken down into sexual and nonsexual forms of violence, which often co-occur with one another (Grych & Swan, 2012).

In addition, preliminary research has associated CDA with maladaptive personality traits, negative emotionality, and impulsivity (Biolcati et al., 2022; Linares et al., 2021; Pineda et al., 2021), which is consistent with relationship violence research (Curtis et al., 2023; Maloney et al., 2024; Sesar et al., 2015). Maladaptive personality traits pertain to extreme negative levels of personality constructs (i.e., psychopathy, Machiavellianism, and narcissism; Dilchert et al., 2014; Wissing & Reinhard, 2017), while impulsivity is characterized by a lack of restraint over behavioral urges (DeYoung & Rueter, 2010). The relationship violence literature may inform these expected associations with CDA, given the noticeable dearth of CDA literature and that the psychological harm caused by CDA contributes to adverse mental health outcomes even after controlling for other forms of violence (Mechanic et al., 2008). However, despite some similarities, CDA and relationship violence are distinct (Pineda et al., 2021), and CDA should be investigated as its own rapidly growing construct.

### Maladaptive Personality Traits

Past studies have found that maladaptive personality traits may be related to CDA among men and women (de Jesus Costa et al., 2023). Specifically, psychopathy and narcissism may be related to perpetration (Branson & March, 2021; Pineda et al., 2021), while sadism and narcissism may be related to victimization (Pineda et al., 2021), likely due to impulsivity and emotional reactivity associated with these characteristics (Branson & March, 2021; Pineda et al., 2021). This is consistent with the relationship violence literature in that maladaptive traits have been consistently associated with perpetration (Maloney et al., 2024; Riaz et al., 2021). However, some associations with relationship violence may not seamlessly extend to CDA, as one study found that CDA perpetration was associated with psychopathy and narcissism but not Machiavellianism, or manipulativeness toward others, for both men and women (Paulhus & Williams, 2002; Pineda et al., 2021). These differences demonstrate how the relationships between personality traits and relationship violence may differ from those between personality traits and other violent behaviors (Walsh et al., 2007), including CDA.

### Negative Emotionality

In addition to maladaptive personality traits, CDA among men and women is also strongly associated with negative emotionalities such as depression, anxiety, and stress (Cava et al., 2020; Lu et al., 2018; Tarriño-Concejero et al., 2023; Toplu-Demritaş et al., 2020). For example, one study showed that CDA victimization was associated with depression and anxiety, while perpetration was associated with stress for both men and women (Tarriño-Concejero et al., 2023). Although prior studies have attempted to determine temporality in the association between CDA victimization and negative emotionality (Cava et al., 2020; Lu et al., 2018; Toplu-Demritaş et al., 2020), these findings have been largely inconsistent.

The relationship violence literature yields stronger support for depression predicting later victimization (Edwards et al., 2006; Goldstein et al., 2008; Keenan-Miller et al., 2007). Indeed, those with negative emotionality may be more susceptible to abuse and manipulation within romantic relationships. That negative emotionality is also associated with the perpetration of relationship violence among men and women (Sesar et al., 2015; Ulloa et al., 2014) indicates that those who experience more attachment anxiety and depression may be more likely to externalize these feelings in unhealthy ways, such as through perpetration (Ulloa et al., 2014; Yu et al., 2018). These interpretations may extend to CDA in that individuals with mental health vulnerabilities may be targeted by perpetrators (Hinduja & Patchin, 2020), possibly as a form of control or externalization of negative emotionality.

### Impulsivity

Furthermore, CDA has also been studied within the context of impulsivity, which may be comprised of various facets (e.g., attentional, motor, and nonplanning). The few studies investigating impulsivity’s role in CDA have observed that men and women with greater emotional instability and impulsivity were more likely to be victimized and perpetrate CDA (Linares et al., 2021). These findings are congruent with prior literature, which identified a direct effect of impulsivity on CDA victimization (Álvarez-García et al., 2019) and research showing that impulsive and emotionally unstable individuals may be more likely to perpetrate CDA (Biolcati et al., 2022; Branson & March, 2021). Those who are more impulsive may be more reactive toward their partner and engage in CDA perpetration (Linares et al., 2021; Sparks et al., 2023), while those who impulsively post online may be easily accessed and victimized by perpetrators (Van Ouytsel et al., 2018). Indeed, relationship violence and impulsivity are also strongly related to one another (Leone et al., 2016; Miller et al., 2012), with one study finding impulsivity related to different forms of relationship violence (Curtis et al., 2023). Although research shows that general impulsivity is associated with CDA, how different aspects of impulsivity may relate to CDA has not been investigated.

### Present Study

Despite similarities with relationship violence, CDA remains understudied and merits further investigation to determine factors that may predispose individuals to perpetrate. Specifically, the limited, extant literature investigating CDA suggests that a culmination of background factors such as personality, negative emotionality, and impulsivity may all contribute to one’s propensity for CDA victimization or perpetration (Biolcati et al., 2022; Linares et al., 2021; Pineda et al., 2021). Further, given that past research has noted limited gender differences regarding the relationships between these background characteristics and CDA, we did not hypothesize differences between men and women. Moreover, while CDA has yet to be investigated across age groups, the prevalence of relationship violence peaks within 18- to 24-year-olds and decreases within older adults (Thompson et al., 2006). Thus, these relationships were specifically investigated using a college student sample. The present study assessed whether profiles comprised of maladaptive personality, negative emotionality, and impulsivity may be more strongly associated with CDA victimization and/or perpetration. Based on the extant CDA and relationship violence literature, the authors hypothesized (a) maladaptive personality, negative emotionality, and impulsivity would be related to both CDA victimization and perpetration; (b) participants could be clustered into distinct profiles based on these background characteristics; and (c) these profiles would be associated with differences in CDA victimization and perpetration. Specifically, profiles lower in aversive personality traits and impulsivity would be least likely to perpetrate or be victimized, while those with higher impulsivity and greater negative emotionality would be most likely to be victimized or to perpetrate.

Finally, because extant research was conducted primarily in general populations, a secondary goal of this study was to explore whether CDA may manifest itself in similar ways based on personality, negative emotionality, and impulsivity characteristics among a primarily Latinx population relative to the more general populations assessed in previous research. Differences in manifestations of CDA may be present, as past studies have found that cultural factors may contribute to CDA within Latinx populations. Specifically, Latinx individuals report greater tolerance for dating violence, particularly psychological violence (Villagrán et al., 2024). Further, Mexican American values such as machismo may impact behaviors, attitudes, and tolerance for relationship violence (Terrazas-Carrillo & Sabina, 2019). Machismo is the expectation in Latinx culture that men should protect their families, be chivalrous, and be family centered; however, other presentations of this value include the normalization of aggression, sexism, and chauvinism (Arciniega et al., 2008). Thus, machismo beliefs may be associated with general dating violence (Terrazas-Carrillo & Sabina, 2019) as well as surveillance behaviors (Terrazas-Carrillo et al., 2023) similar to those of CDA. Hence, the authors aim to explore whether CDA manifests itself within a Latinx population similar to more general samples given these cultural differences between Latinx and non-Latinx populations.

## Methods

### Participants

A total of 590 18- to 30-year-old college students from a Hispanic Serving Institution (*M*_age_ = 20.35, *SD* = 2.48, 75.6% female) participated in the present study. Participants were recruited through SONA, a university web-based recruitment system. Respondents were predominantly Hispanic/Latinx (85.3%), 8.1% identified as White/Caucasian, 4% as Black/African American, 1.4% as Asian/ Pacific Islander, and 0.9% as other. Furthermore, 11 participants failed more than 1 out of 6 attention checks, leaving a final sample of 579 for data analysis.

### Procedure

University IRB approval was obtained prior to data collection, and participants were recruited for a larger study on “personality and behavioral associations with addictive behaviors.” Interested participants provided informed consent and completed questionnaires on Qualtrics (2024). Subsequently, participants were debriefed and received research participation credit. Data were collected from September 2020 to May 2021. Data are available at: 10.6084/m9.figshare.19216899

### Measures

#### Cyber Dating Abuse Victimization and Perpetration Scale

The Cyber Dating Abuse Victimization and Perpetration Scale was utilized to assess four dimensions of online relational abuse: sexual victimization, nonsexual victimization, sexual perpetration, and nonsexual perpetration (Zweig et al., 2013). This measure contained 32 items answered on a 4-point Likert scale (0 = “*Never*” to 3 = “*Very often*”) in which sums are obtained for each respective subscale, with higher scores indicating greater CDA. The scale demonstrated acceptable reliability across subscales in the present study (α = .82–.98).

#### Short Dark Triad

The Short Dark Triad (Jones & Paulhus, 2014) measures three aversive personality traits: narcissism, Machiavellianism, and psychopathy. This self-report measure is comprised of 27 items answered on a 5-point Likert scale (1 = “*strongly disagree*” to 5 = “*strongly agree*”) in which subscale scores are calculated using an average and higher scores indicating greater levels of personality traits. This scale demonstrated low to acceptable reliability for the narcissism (α = .66) Machiavellianism (α = .79), and psychopathy (α = .72) subscales in the present study.

#### Depression, Anxiety, and Stress Scale

The Depression, Anxiety, and Stress Scale (DASS-21; Lovibond & Lovibond, 1995) assesses three mental health components: depression, anxiety, and stress over a weekly time frame. This self-report measure is comprised of 21 items answered on a 4-point Likert scale (0 “*Did not apply to me at all*” to 3 = “*Applied to me very much, or most of the time*”) in which items are summed and multiplied by 2 to obtain a subscale score with higher values indicating greater negative emotionality. Cut-off scores for levels of depression (<6: subclinical, 7–8: mild, 9–13: moderate, 14–16: severe, >17: extremely severe), anxiety (<5: subclinical, 6–7: mild, 8–12: moderate, 13–15: severe, >16: extremely severe), and stress (<11: subclinical, 12–13: mild, 14–16: moderate, 17–18: severe, >19: extremely severe) are determined by the clinical recommendations outlined in the scoring procedures of the DASS-21 (Szabo & Lovibond, 2022). This scale demonstrated excellent reliability (α = .87–.92).

#### Barratt Impulsiveness Scale

The Barratt Impulsiveness Scale (Patton et al., 1995) is a 30-item self-report measure assessing three dimensions of impulsivity: attentional, motor, and nonplanning. Items answered on a 4-point Likert scale (1 = “*rarely/never*” to 4 = “*almost always/always*”) in which scores are summed and higher values indicate greater levels of impulsivity. This scale demonstrated low to acceptable reliability for the attentional (α = .67), motor (α = .68), and nonplanning (α = .72) subscales in the present study.

## Results

### Preliminary Analyses

Skewness and kurtosis for all predictor variables were assessed, and all independent variables fell within the recommended range of |SK| < 2 and |KU| < 7 (Kim, 2013); however, all of the CDA outcome variables were positively skewed, and yielded kurtosis values outside the recommended range (see Table 1). Transformations were attempted to normalize these variables, yet they continued to violate assumptions of normality, so researchers opted for nonparametric tests (e.g., Kruskal–Wallis; Wadgave & Khairnar, 2019).

**Table 1.** Correlation Matrix, Means, Standard Deviations, Skewness, and Kurtosis.

Variable	1.	2.	3.	4.	5.	6.	7.	8.	9.	10.	11.	12.	13.
Cyber Dating Abuse													
1. Sexual Vic	1												
2. Nonsexual Vic	**.55**	1											
3. Sexual Perp	**.39**	**.65**	1										
4. Nonsexual Perp	**.36**	**.67**	**.92**	1									
Impulsivity													
5. Attentional	**.12**	.08	.07	**.10**	1								
6. Motor	**.10**	**.14**	**.16**	**.17**	**.44**	1							
7. Nonplanning	.07	.04	.03	.06	**.44**	**.47**	1						
Short Dark Triad													
8. Machiavellianism	**.16**	**.16**	**.11**	**.10**	**.20**	**.23**	**.11**	1					
9. Psychopathy	**.20**	**.21**	**.21**	**.22**	**.24**	**.38**	**.26**	**.52**	1				
10. Narcissism	.06	**.09**	**.09**	**.10**	−.02	**.18**	.01	**.27**	**.32**	1			
DASS													
11. Depression	**.18**	**.15**	**.12**	**.14**	**.42**	**.19**	**.21**	**.19**	**.21**	−**.13**	1		
12. Anxiety	**.23**	**.20**	**.15**	**.17**	**.44**	**.24**	**.13**	**.15**	**.20**	.01	**.72**	1	
13. Stress	**.23**	**.18**	**.13**	**.15**	**.48**	**.22**	**.15**	**.19**	**.19**	−.04	**.80**	**.83**	1
Mean	0.29	0.09	0.04	0.04	17.64	21.54	24.10	2.89	2.17	2.84	13.42	11.54	14.57
Standard deviation	0.55	0.30	0.23	0.25	3.95	4.71	5.13	0.67	0.63	0.54	11.67	10.26	10.62
Skewness	2.06	4.96	8.63	8.05	0.38	0.62	−0.03	0.01	0.21	−0.06	0.66	0.88	0.38
Kurtosis	3.67	30.45	83.55	69.54	−0.11	0.21	−0.56	0.23	−0.41	0.50	−0.58	0.02	−0.72

*Note*. Bold indicates significance at *p* < .05. DASS = Depression, Anxiety, and Stress scale.

Within this sample, 31.5% of participants indicated having been victims of sexual CDA, 21.8% having been victims of nonsexual CDA, 4.3% perpetrating sexual CDA, and 7.6% perpetrating nonsexual CDA. Bivariate correlations, means, and standard deviations were also assessed for all study variables (see Table 1).

### Latent Profile Analysis

MPlus Version 8.10 was utilized to generate profiles based on the background characteristics of participants (i.e., Machiavellianism, psychopathy, narcissism, depression, anxiety, and impulsivity). Researchers systematically analyzed two through nine-profile solutions and evaluated model fit indices using the Akaike information criterion (AIC), Bayesian information criterion (BIC), and entropy values (Table 2). According to Ferguson et al. (2020), model fit may be determined by the model resulting in the lowest AIC and BIC values. Similarly, model fit may also be determined using the model with the highest entropy value with acceptable model fit being indicated by those with an entropy value above 0.80 (Tein et al., 2013). According to model results, the four-profile solution seemed to fit the data the best as indicated by a peak in entropy (0.87). However, AIC and BIC values kept decreasing with more subsequent profile solutions, even at the nine-profile solution. Due to the inconclusive results yielded by the AIC and BIC values, the four-profile solution was retained for subsequent analyses.

**Table 2.** Model Fit Statistics.

Model	AIC	BIC	Entropy
Two profiles	25,460.35	25,582.47	0.869
Three profiles	25,111.47	25,277.20	0.870
**Four profiles**	**24,968.66**	**25,178.00**	**0.873**
Five profiles	24,835.86	25,088.81	0.840
Six profiles	24,752.18	25,048.75	0.832
Seven profiles	24,697.01	25,037.19	0.835
Eight profiles	24,635.55	25,019.35	0.839
Nine profiles	24,584.78	25,012.19	0.843

*Note*. Bold Indicates best model fit. AIC = Aikaike’s Information Criterion; BIC = Bayesian Information Criterion.

**Figure 1.**
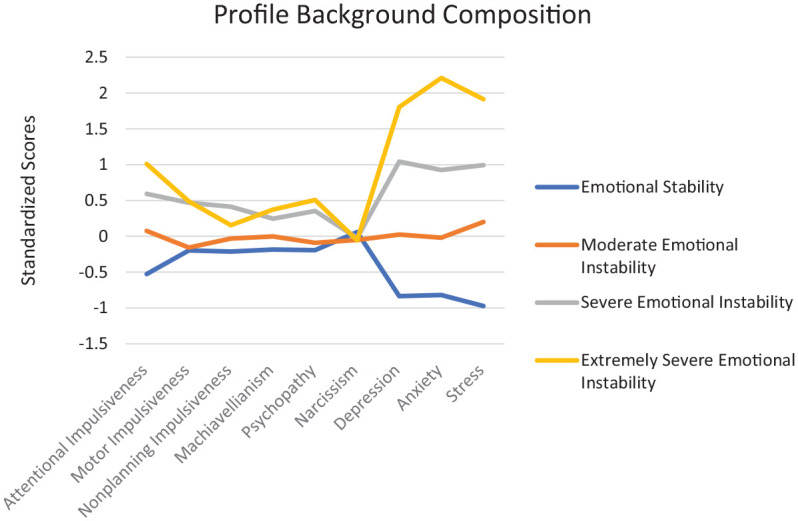
Composition of profile traits.

Figure 1 displays composites of background characteristics for each of the four profiles. Profile 1 (*n* = 236) was characterized by moderate Machiavellianism, psychopathy, and narcissism as well as low-to-moderate attentional, nonplanning, and motor impulsivity. Furthermore, this profile was characterized by subclinical levels of depression (*M* = 3.67), anxiety (*M* = 3.14), and stress (*M* = 4.23) as indicated by cut-off scores (Depression: <5, Anxiety: <5, Stress: <11; Szabo & Lovibond, 2022). Thus, this profile was named “Emotional Stability” (ES). Profile 2 (*n* = 184) was characterized by moderate levels of maladaptive personality traits, depression, anxiety, stress, and impulsivity, in which scores tended to cluster around the mean for the overall sample. Furthermore, this profile met the criteria for clinically moderate to severe levels of depression (*M* = 13.71), anxiety (*M* = 11.34), and stress (*M* = 16.70) as characterized by cut-off scores (Depression: 9–16, Anxiety: 8–15, Stress: 14–18; Szabo & Lovibond, 2022); thus, this profile was named “Moderate Emotional Instability” (MEI). Profile 3 (*n* 121) was characterized by average narcissism, slightly elevated Machiavellianism and psychopathy, as well as slightly elevated impulsivity across dimensions. Moreover, those in profile 3 met clinical thresholds for extremely severe depression (*M* *=* 25.58), anxiety (*M* *=* 21.04), and stress (*M* *=* 25.12; Depression: >17, Anxiety: >16, Stress: >19; Szabo & Lovibond, 2022) thus this profile was named “Severe Emotional Instability” (SEI). Lastly, profile 4 (*n* = 38) was characterized by moderate levels of narcissism and nonplanning impulsivity, and higher levels of Machiavellianism, psychopathy, attentional, and motor impulsivity. These individuals also met clinical thresholds for extremely severe depression (*M* *=* 34.49), anxiety (*M* *=* 34.21), and stress (*M* 34.89; Depression: >17, Anxiety: >16, Stress: >19; Szabo & Lovibond, 2022); however, their scores were roughly 1 standard deviation higher than those in the SEI profile, on average. Thus, this profile was named “Extremely Severe Emotional Instability” (ESEI).

**Figure 2.**
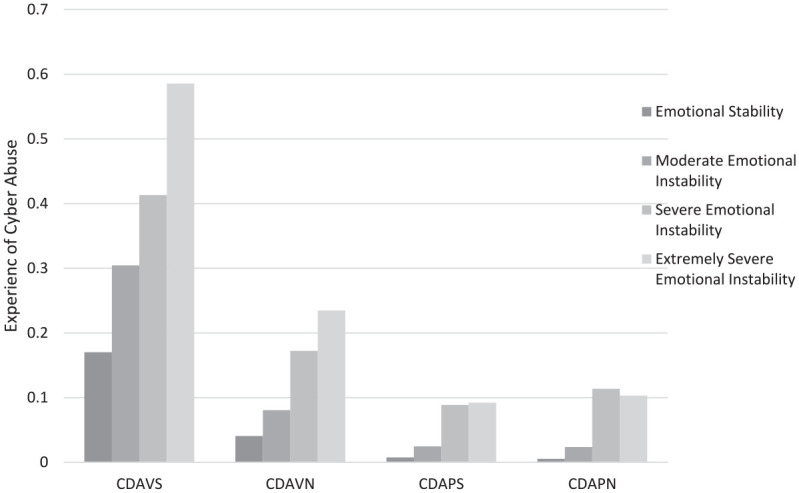
Associations between profile membership and experiences of cyber abuse. *Note.* CDAVS = cyber dating victimization-sexual; CDAVN = cyber dating victimization-nonsexual; CDAPS = cyber abuse perpetration-sexual; CDAPN = cyber abuse perpetration-nonsexual.

SPSS Version 29.0.2 (IBM Corp., 2024) was utilized to conduct four Kruskal–Wallis one-way analyses of variance tests and determine the association between profile and sexual CDA victimization, nonsexual CDA victimization, sexual CDA perpetration, and nonsexual CDA perpetration (Figure 2). Results revealed that profile membership predicted sexual CDA perpetration (*χ*^2^(3) = 8.804, *p* = .032), such that those in the “ES” profile reported less sexual CDA perpetration than those in the “SEI” profile (*p* = .006). However, there were no statistical differences between the remaining profiles.

Profile membership predicted nonsexual CDA perpetration (χ^2^(3) = 17.012, *p* < .001), and post hoc pairwise comparisons indicated that individuals in the “ES” profile reported less nonsexual CDA perpetration than those in the “SEI” (*p* < .001) and the “ESEI” profiles (*p* = .005). There were no statistical differences between the remaining profiles.

Profile membership also predicted sexual CDA victimization (χ^2^(3) = 25.616, *p* < .001). Furthermore, a post hoc pairwise comparison test indicated that individuals in the “ES” profile reported less sexual CDA victimization than those in the “MEI” profile (*p* = .003), the “SEI” profile (*p* < .001), and the “ESEI” profile (*p* < .001). There were no statistical differences between those in the “MEI,” “SEI,” and “ESEI” profiles.

Lastly, profile membership also predicted nonsexual CDA victimization (χ^2^(3) = 35.093, *p* < .001), and post hoc comparisons showed that individuals in the “ES” profile reported less nonsexual CDA victimization than those in the “SEI” (*p* < .001) and “ESEI” profiles (*p* < .001). Likewise, those in the “MEI” profile also reported less nonsexual CDA victimization than those in “SEI” (*p* = .008) and the “ESEI” profiles (*p* = .004). However, there were no statistical differences between the “SEI” and “ESEI” profiles or between the “ES” and “MEI” profiles.

## Discussion

The present study assessed how maladaptive personality traits (i.e., Machiavellianism, psychopathy, and narcissism), depression, anxiety, stress, and impulsivity may be related to CDA perpetration and victimization and whether CDA among Latinx individuals may manifest itself similar to general populations noted in prior studies. Moreover, the present study investigated whether these characteristics could be categorized into distinct profiles predicting differences in reported CDA perpetration and victimization. Our first hypothesis was supported, such that these background characteristics were associated with CDA perpetration and victimization. Furthermore, these background characteristics comprised four distinct profiles in which participants reported differing rates of CDA perpetration and victimization based on profile membership, thus supporting our second and third hypotheses. Finally, factors associated with CDA victimization and perpetration in prior studies also demonstrated similar associations among a predominantly Latinx sample, suggesting that the predictors of CDA among predominantly Latinx populations may be similar to those among more general populations.

Our findings suggest that individuals in the “SEI” and “ESEI” profiles are most likely to report CDA perpetration and victimization. This may be due to impulsive individuals’ sensitivity to negative emotional states, as past studies have found this combination positively associated with CDA perpetration and victimization (Linares et al., 2021). Moreover, the broad variability of depression, anxiety, and stress was the main distinguishing factor between the four profiles, though differences in impulsivity and maladaptive personality traits also accompanied this negative emotionality. This stark variability in negative emotionality may have been impacted by COVID-19 due to the increases in negative mental health outcomes during COVID-19 lockdowns (Elmer et al., 2020).

### Sexual Perpetration

Those in the “ES” profile displayed the lowest propensity to perpetrate sexual CDA when compared to those in the “SEI” profile, as the “ES” profile was characterized, in part, by subclinical levels of depression, anxiety, and stress, while those in the “SEI” profile reported extremely severe levels of depression, anxiety, and stress. These findings are congruent with past studies suggesting that higher levels of negative emotionality are associated with more frequent CDA perpetration (Tarriño-Concejero et al., 2023). Specifically, those who experience greater depression, anxiety, and stress may be more controlling and aggressive to satisfy their desire for reassurance from their partner. Indeed, past research on relationship violence found negative emotionality to predict more frequent sexual coercion against a partner when that partner tries to avoid having sex (Brassard et al., 2007). Similarly, those with lower levels of negative emotions in the “ES” profile may have a lower propensity to perpetrate sexual CDA as they do not need to gain reassurance from their partner.

Moreover, these profiles were further differentiated by lower levels of Machiavellianism, psychopathy, and impulsivity. This is supported by past research associating maladaptive personality traits and negative emotionality with greater perpetration of sexual and nonsexual CDA (Linares et al., 2021; Smoker & March, 2017; Sparks et al., 2023; Tarriño-Concejero et al., 2023). Specifically, those with greater maladaptive personality traits and greater impulsivity may be more likely to seek reassurance by threatening their partner (Duncan & March, 2019; Laforte et al., 2023; Smoker & March, 2017). In contrast, those in the “ES” profile may be better able to regulate their emotions and manage their impulses than those in the “SEI” profile, making them less likely to respond in a sexually threatening way to their partner (Linares et al., 2021; Sparks et al., 2023). Furthermore, although individuals in the “ESEI” profile did not report greater rates of sexual perpetration while those in the “SEI” profile did, this difference may have been due to the low rates of CDA perpetration and small sample size in the “ESEI” profile, which may have resulted in a lack of power. Future research should seek to examine this relationship more closely to affirm this explanation of these results.

### Nonsexual Perpetration

The “ES” profile was associated with the lowest propensity to perpetrate nonsexual CDA when compared to the “SEI” and “ESEI” profiles, possibly due to the lower levels of depression, anxiety, and stress associated with the “ES.” Specifically, greater depression, anxiety, and stress are associated with more frequent CDA perpetration (Tarriño-Concejero et al., 2023; Zweig et al., 2013), and past research on relationship violence has suggested that the internal distress associated with such emotional states may lead to the usage of aggressive or controlling behaviors toward one’s partner to regain a sense of self-worth and control (Ulloa et al., 2014). Hence, those in the “ES” profile were less likely to perpetrate than those in the “SEI” and “ESEI” profiles, suggesting “ES” individuals may not externalize internal turmoil as CDA perpetration, due to their lower levels of emotional distress.

Furthermore, those in the “SEI” and “ESEI” profiles report greater impulsivity and maladaptive personality traits, which may result in loss of control in response to their partner provoking feelings of anxiety or anger and to engage in controlling mechanisms to reassert dominance and control over their partner’s behaviors (Linares et al., 2021; Pineda et al., 2021). This negative emotionality may exacerbate the expression of impulsive behaviors by functioning as a catalyst for an impulsive behavioral response that results in an individual engaging in CDA perpetration toward their partner (Linares et al., 2021). This supports a prior study that noted that individuals classified in the high propensity for dating and peer violence group had greater rates of depression and anxiety than those in the least violent group (Garthe et al., 2021). Therefore, those in the “ES” profile may experience less intense negative emotionality and have greater control over their impulses in response to such states, making them less likely to engage in nonsexual CDA perpetration than those in the “SEI” and “ESEI” profiles. Alternatively, cultural factors such as marianismo and machismo may have also influenced this association in this primarily Latinx sample, given that CDA may consist of controlling behaviors that are normalized (Litz & Holvoet, 2021); however, these variables were not specifically assessed in the present study, so prospective studies should more closely assess this possible association.

### Sexual Victimization

Similar to perpetration, those in the “ES” profile reported fewer experiences of sexual CDA victimization than all other profiles, which may also be largely due to profile differences in negative emotionality. Past studies have noted that those with greater emotional regulation skills are less likely to use sexting to help them regulate their emotions (Trub & Starks, 2017) making them less likely to be victimized by perpetrators who target those engaging in sexting (Gassó et al., 2019). Indeed, greater negative emotionality has been associated with nonconsensual sexual CDA experiences, such as the dissemination of sexual content, and coercion into sending sexually explicit photographs and/or content (Gasso et al., 2019). Those in the “ES” profile may be less likely to disseminate sexual content online, resulting in them being less likely to experience online sexual victimization (Linares et al., 2021). Further, those in this profile may have greater emotional regulation capabilities as they reported lower levels of negative emotionality and impulsivity (Trub & Starks, 2017).

In addition to negative emotionality, impulsivity, and maladaptive personality traits have also been associated with greater rates of sexual CDA victimization in adolescents (Gassó et al., 2019; Hernández et al., 2021), especially when co-occurring with negative emotionality (Hernández et al., 2021; Linares et al., 2021). This suggests that those in more reactive profiles such as “MEI,” “SEI,” and “ESEI” may impulsively use sexual messages to try and attain validation from a romantic partner when experiencing unpleasant emotions (Hernández et al., 2021; Linares et al., 2021). However, these intentions may be misconstrued or taken advantage of by partners, who may misappropriate explicit images or request sexual favors rather than providing validation. This interpretation is congruent with past studies showing that social rejection contributed to increased online sexual engagement and experiencing nonconsensual distribution of sexual content, which further aggravated negative emotionality (Gassó et al., 2019; Hernandez et al., 2021). Thus, those in the “ES” profile may be less likely to seek validation through online sexual communication, making them less likely to experience sexual CDA than individuals in the other profiles.

### Nonsexual Victimization

The “SEI” and “ESEI” profiles differed significantly from the other profiles based on their levels of negative emotionality, which has been associated with nonsexual relationship violence victimization (Iverson et al., 2022; Sandberg et al., 2019). Specifically, negative emotionality may decrease the cognitive and affective capacity to identify potential abusers and make decisions to avoid risks or violent triggers of a partner (Iverson et al., 2022). This may result in long-lasting emotional states (i.e., hopelessness, worthlessness, and low motivation/energy) and difficulties terminating these relationships (Iverson et al., 2022). In other words, those in the “ES” and “MEI” profiles may be less likely to have experienced the lasting effects of depression, anxiety, and stress, and be more likely to detect signs of nonsexual CDA victimization and to terminate abusive online relationships.

These findings are also congruent with literature suggesting that victimization is associated with greater levels of maladaptive personality traits (Pineda et al., 2021), depression, anxiety, stress (Tarriño-Concejero et al., 2023), and impulsivity (Álvarez-García et al., 2019). Past studies have found associations between time spent online and both negative emotionality (Labrague, 2014) and CDA victimization (Van Ouytsel et al., 2018) which suggest that having a large online presence may present potential perpetrators with greater access to those they victimize. Indeed, those who experience negative emotionality and poor impulse control may be more likely to post online (Turel & Qahri-Saremi, 2018) and become targets of nonsexual CDA. In addition, those with poor impulse control tend to engage in addictive internet behaviors, which may predispose them to experiencing direct CDA (Linares et al., 2021). In other words, those in the “SEI” and the “ESEI” may be more likely to experience nonsexual CDA victimization as they may have difficulties regulating their impulses and engage with online content more frequently, especially when experiencing negative emotionality. However, future studies should investigate these relationships and their underlying mechanism more closely.

### Limitations

The present study contains several notable limitations. Reported CDA perpetration rates were low compared to previous studies utilizing a similar sample (Curry & Zavala, 2020), which may have resulted in a lack of statistical power to detect the relevant differences between profiles when assessing perpetration, especially with regards to the “ESEI” profile, which included the fewest participants. Furthermore, this study was cross-sectional, so causal inferences cannot be made about profile membership and future perpetration and/or victimization. Moreover, based on the function of the Latent Profile Analysis, some participants may have reported characteristics that could have suggested categorization into two different profiles; however, this pattern of responses was not common enough to suggest the addition of a fifth profile. Lastly, although the present study’s results suggest that the predictors of CDA perpetration and victimization are similar in predominantly Latinx populations compared to more general populations, the sample was also predominantly female and college-aged, potentially limiting the generalizability of the present findings to other populations.

### Future Directions

Findings from the present study warrant further investigation to explore the four-profile solution with other samples, including criminal populations given that justice-involved persons may display higher levels of maladaptive personality traits and impulsivity (Tharshini et al., 2021). Furthermore, these findings should also be replicated in samples with a more equal distribution of gender as some past studies have noted gender differences in CDA (Biolcati et al., 2022). In addition, given that the sample was predominately Latinx, cultural factors (i.e., marianismo and machismo) may have contributed to a normalization of dating violence (Rivera et al., 2024; Terrazas-Carrillo et al., 2023) and should be examined in future studies. Lastly, as prior studies have noted the impact of online activity on experiences of CDA (Van Ouytsel et al., 2018) prospective studies may want to investigate associations with time spent online.

### Clinical Implications

Overall, participants in the present study displayed a wide variability of depression, anxiety, and stress scores with those in the “Severe Emotional Instability” and “Extremely Severe Emotional Instability” profiles meeting extremely severe thresholds (Szabo & Lovibond, 2022). Furthermore, there were greater rates of reported victimization with this sample compared to past studies on CDA (Víllora et al., 2021), possibly due to the increased online presence of many individuals during the COVID-19 lockdown, making them more susceptible to victimization. Given that social media usage remains elevated (Gottfried, 2024), individuals in these more reactive profiles may remain at a greater risk for future victimization. Results from the present study may also suggest that past predictors of CDA in general samples generalize well to predominantly Latinx samples. Future interventions should consider including emotion regulation skills and positive coping strategies to reduce feelings of depression, anxiety, and stress, to mitigate possible perpetration and victimization.

## Conclusions

The present study extends our nascent understanding of characteristics contributing to CDA perpetration and victimization among predominantly Latinx individuals. Understanding the profile makeup of those more likely to experience CDA is important given the increased rates of CDA during the COVID-19 lockdown (Huiskes et al., 2022; Sheridan-Johnson et al., 2024) and the greater prevalence of online relationships (Gottfried, 2024). Consistent with past research (de Jesus Costa et al., 2023), those in profiles who scored lower in maladaptive personality traits, depression, anxiety, stress, and impulsivity displayed the lowest amount of perpetration and victimization, suggesting that interventions targeting these key variables may be beneficial to reduce rates of both perpetration and victimization. Further studies are warranted to replicate and extend these findings as CDA rates remain elevated post-COVID-19 and are detrimental to individuals’ well-being.

## References

[bibr1-08862605251339636] Álvarez-García D. Núñez J. C. González-Castro P. Rodríguez C. Cerezo R. (2019). The effect of parental control on cyber-victimization in adolescence: The mediating role of impulsivity and high-risk behaviors. Frontiers in Psychology, 10, 1159. 10.3389/fpsyg.2019.01159 31178790 PMC6538814

[bibr2-08862605251339636] Biolcati R. Pupi V. Mancini G. (2022). Cyber dating abuses and ghosting behaviors: Personality and gender roles in romantic relationships. Current Issues in Personality Psychology, 10(3), 240–251. 10.5114/cipp.2021.108289 38013819 PMC10535627

[bibr3-08862605251339636] Branson M. March E . (2021). Dangerous dating in the digital age: Jealousy, hostility, narcissism, and psychopathy as predictors of cyber dating abuse. Computers in Human Behavior, 119, 106711. 10.1016/j.chb.2021.106711

[bibr4-08862605251339636] Brassard A. Shaver P. R. Lussier Y. (2007). Attachment, sexual experience, and sexual pressure in romantic relationships: A dyadic approach. Personal Relationships, 14(3), 475–493. 10.1111/j.1475-6811.2007.00166.x

[bibr5-08862605251339636] Cava M.-J. Tomás I. Buelga S. Carrascosa L. (2020). Loneliness, depressive mood and cyberbullying victimization in adolescent victims of Cyber Dating Violence. International Journal of Environmental Research and Public Health, 17(12), 4269. 10.3390/ijerph17124269 32549276 PMC7345753

[bibr6-08862605251339636] Curry T. R. Zavala E. (2020). A multi-theoretical perspective on cyber dating abuse victimization and perpetration within intimate relationships: A test of general strain, social learning, and self-control theories. Victims & Offenders, 15(4), 499–519.

[bibr7-08862605251339636] Curtis A. Harries T. Bereznicki H. Skvarc D. Patafio B. Hyder S. Mayshak R. (2023). Facet-level impulsivity and proactive and reactive relational aggression. Personality and Individual Differences, 213, 112320. 10.1016/j.paid.2023.112320

[bibr8-08862605251339636] de Jesus Costa B. Simoes A. M. Carvalho Revla I . (2023). Psychopathic traits and cyber dating abuse: Mediating effect of internet addiction in a university student sample. Journal of Forensic Psychology Research and Practice, 23(2), 113–135. 10.1080/24732850.2021.2016116

[bibr9-08862605251339636] DeYoung C. G. Rueter A. R. (2010). Impulsivity as a personality trait. Handbook of Self-Regulation: Research, Theory, and Applications, 2, 485–502.

[bibr10-08862605251339636] Dilchert S. Ones D. S. Krueger R. F. (2014). Maladaptive personality constructs, measures, and work behaviors. Industrial and Organizational Psychology: Perspectives on Science and Practice, 7(1), 98–110. 10.1111/iops.12115

[bibr11-08862605251339636] Duncan Z. March E. (2019). Using Tinder® to start a fire: Predicting antisocial use of Tinder® with gender and the Dark Tetrad. Personality and Individual Differences, 145, 9–14. 10.1016/j.paid.2019.03.014

[bibr12-08862605251339636] Edwards M. E. Green C. Perkins U. E. (2006). Teen dating violence, ethnic identity and depression in inner city African American youths and young adults. Journal of Knowledge and Best Practices in Juvenile Justice and Psychology, 1(1), 41–50.

[bibr13-08862605251339636] Elmer T. Mepham K. Stadtfeld C. (2020). Students under lockdown: Comparisons of students’ social networks and mental health before and during the COVID-19 crisis in Switzerland. PLoS One, 15(7), e0236337. 10.1371/journal.pone.0236337 PMC7377438 32702065

[bibr14-08862605251339636] Ferguson S. L. G. Moore E. W. Hull D. M. (2020). Finding latent groups in observed data: A primer on latent proﬁle analysis in Mplus for applied researchers. International Journal of Behavioral Development, 44(5), 458–468. 10.1177/0165025419881721

[bibr15-08862605251339636] Garthe R. C. Sullivan T. N. Behrhorst K. L. (2021). A Latent Class Analysis of early adolescent peer and dating violence: Associations with symptoms of depression and anxiety. Journal of Interpersonal Violence, 36(5–6), 2031–2049. 10.1177/0886260518759654 29475422

[bibr16-08862605251339636] Gassó A. M. Klettke B. Agustina J. R. Montiel I. (2019). Sexting, mental health, and victimization among adolescents: A literature review. International Journal of Environmental Research and Public Health, 16(13), 2364. 10.3390/ijerph16132364 31277335 PMC6650829

[bibr17-08862605251339636] Goldstein S. E. Chesir-Teran D. McFaul A. (2008). Profiles and correlates of relational aggression in young adults’ romantic relationships. Journal of Youth and Adolescence, 37(3), 251–265. 10.1007/s10964-007-9255-6

[bibr18-08862605251339636] Gottfried J. (2024, January 31). Americans’ social media use. Pew Research Center. https://www.pewresearch.org/internet/2024/01/31/americans-social-media-use/

[bibr19-08862605251339636] Grych J. Swan S. (2012). Toward a more comprehensive understanding of interpersonal violence: Introduction to the special issue on interconnections among different types of violence. Psychology of Violence, 2(2), 105–110. 10.1037/a0027616

[bibr20-08862605251339636] Hernández M. P. Schoeps K. Maganto C. Montoya-Castilla I. (2021). The risk of sexual-erotic online behavior in adolescents—Which personality factors predict sexting and grooming victimization? Computers in Human Behavior, 114, 106569. 10.1016/j.chb.2020.106569

[bibr21-08862605251339636] Hinduja S. Patchin J. W. (2020). Digital dating abuse among a national sample of U.S. youth. Journal of Interpersonal Violence, 36(23–24), 11088–11108. 10.1177/0886260519897344 31910725

[bibr22-08862605251339636] Huiskes P. Dinis M. A. P. Caridade S. (2022). Technology-facilitated sexual violence victimization during the COVID-19 pandemic: Behaviors and attitudes. Journal of Aggression, Maltreatment & Trauma, 31(9), 1148–1167.

[bibr23-08862605251339636] IBM Corp. (2024). IBM SPSS Statistics for Windows (Version 29.0) [Statistical software]. IBM Corp. https://www.ibm.com/products/spss-statistics

[bibr24-08862605251339636] Iverson K. M. Rossi F. S. Nillni Y. I. Fox A. B. Galovski T. E. (2022). PTSD and depression symptoms increase women’s risk for experiencing future intimate partner violence. International Journal of Environmental Research and Public Health, 19(19), 12217. 10.3390/ijerph191912217 36231518 PMC9566456

[bibr25-08862605251339636] Johnson W. L. Giordano P. C. Manning W. D. Longmore M. A. (2015). The age–IPV curve: Changes in the perpetration of intimate partner violence during adolescence and young adulthood. Journal of Youth and Adolescence, 44(3), 708–726. 10.1007/s10964-014-0158-z 25081024 PMC4332391

[bibr26-08862605251339636] Jones D. N. Paulhus D. L. (2014). Introducing the Short Dark Triad (SD3): A brief measure of dark personality traits. Assessment, 21(1), 28–41. 10.1177/1073191113514105 24322012

[bibr27-08862605251339636] Keenan-Miller D. Hammen C. Brennan P. (2007). Adolescent psychosocial risk factors for severe intimate partner violence in young adulthood. Journal of Consulting and Clinical Psychology, 75(3), 456–463. 10.1037/0022-006x.75.3.456 17563162

[bibr28-08862605251339636] Kim H. Y. (2013). Statistical notes for clinical researchers: assessing normal distribution (2) using skewness and kurtosis. Restorative Dentistry & Endodontics, 38(1), 52. 23495371 10.5395/rde.2013.38.1.52 PMC3591587

[bibr29-08862605251339636] Labrague L. J. (2014). Facebook use and adolescents’ emotional states of depression, anxiety, and stress. Health Science Journal, 8(1), 80.

[bibr30-08862605251339636] Laforte S. Paradis A. Todorov E. H. Cyr C. (2023). Romantic attachment and cyber dating violence in adolescence: A dyadic approach. Journal of Adolescence, 95(4), 647–660. 10.1002/jad.12141 36659837

[bibr31-08862605251339636] Leone R. M. Crane C. A. Parrott D. J. Eckhardt C. I. (2016). Problematic drinking, impulsivity, and physical IPV perpetration: A dyadic analysis. Psychology of Addictive Behaviors, 30(3), 356–366. 10.1037/adb0000159 26828640 PMC4877202

[bibr32-08862605251339636] Li J. Ran G. Zhang Q. He X. (2023). The prevalence of cyber dating abuse among adolescents and emerging adults: A meta-analysis. Computers in Human Behavior, 144, 107726. 10.1016/j.chb.2023.107726

[bibr33-08862605251339636] Linares R. Aranda M. García-Domingo M. Amezcua T. Fuentes V. Moreno-Padilla M. (2021). Cyber-dating abuse in young adult couples: Relations with sexist attitudes and violence justification, smartphone usage and impulsivity. PLoS One, 16(6), e0253180. 10.1371/journal.pone.0253180 PMC8216513 34153073

[bibr34-08862605251339636] Litz K. Holvoet N. (2021). Adolescent dating violence among Nicaraguan youth. Violence Against Women, 27(2), 167–186. 10.1177/1077801219889182 31814528

[bibr35-08862605251339636] Lovibond P. F. Lovibond S. H. (1995). Depression Anxiety and Stress Scales (DASS-42) [Database record]. APA PsycTests. 10.1037/t39835-000

[bibr36-08862605251339636] Lu Y. Van Ouytsel J. Walrave M. Ponnet K. Temple J. R. (2018). Cross-sectional and temporal associations between cyber dating abuse victimization and mental health and substance use outcomes. Journal of Adolescence, 65(1), 1–5. 10.1016/j.adolescence.2018.02.009 29499572 PMC5932249

[bibr37-08862605251339636] Maloney M. Dashineau S. Massa A. Benefiel C. Eckhardt C. (2024). Dimensions of narcissism and intimate partner aggression: A vignette study. Personality and Individual Differences, 217, 112449. 10.2139/ssrn.4504379

[bibr38-08862605251339636] Mechanic M. Weaver T. Resick P. (2008). Mental health consequences of intimate partner abuse: A multidimensional assessment of four different forms of abuse. Violence Against Women, 14(6) 634–654. 10.1177/1077801208319283 18535306 PMC2967430

[bibr39-08862605251339636] Miller J. D. Zeichner A. Wilson L. F. (2012). Personality correlates of aggression. Journal of Interpersonal Violence, 27(14), 2903–2919. 10.1177/0886260512438279 22610830

[bibr40-08862605251339636] Patton J. H. Stanford M. S. Barratt E. S. (1995). Factor structure of the Barratt Impulsiveness Scale. Journal of Clinical Psychology, 51(6), 768–774. 10.1002/1097-4679(199511)51:6<768::aid-jclp2270510607>3.0.co;2-1 8778124

[bibr41-08862605251339636] Paulhus D. L. Williams K. M. (2002). The dark triad of personality: Narcissism, Machiavellianism, and psychopathy. Journal of Research in Personality, 36(6), 556–563. 10.1016/S0092-6566(02)00505-6

[bibr42-08862605251339636] Peskin M. F. Markham C. M. Shegog R. Temple J. R. Baumler E. R. Addy R. C. Hernandez B. Cuccaro P. Gabay E. K. Thiel M. Emery S. T. (2017). Prevalence and correlates of the perpetration of cyber dating abuse among early adolescents. Journal of Youth and Adolescence, 46(2), 358–375. 10.1007/s10964-016-0568-1 27665278 PMC13011686

[bibr43-08862605251339636] Pineda D. Galán M. Martínez-Martínez A. Campagne D. M. Piqueras J. A. (2021). Same personality, new ways to abuse: How dark tetrad personalities are connected with cyber intimate partner violence. Journal of Interpersonal Violence, 37(13–24), NP11223–NP11241. 10.1177/0886260521991307 33546557

[bibr44-08862605251339636] Qualtrics. (2024). Qualtrics XM – Survey distribution platform (Version 2024) [Computer software]. Qualtrics. https://www.qualtrics.com

[bibr45-08862605251339636] Riaz S. Bano Z. Ahmed I. Naz I. (2021). Predictive relationship of narcissism and psychopathy with relational aggression moderated by age among adolescents. Journal of Ayub Medical College Abbottabad—Pakistan, 33(2), 231–235. 34137535

[bibr46-08862605251339636] Rivera V. P. Cruz A. C. Bonilla G. B. Nieves Y. R. Ortiz A. N. (2024). The psychology of Marianismo: a review of empirical research. Salud Y Conducta Humana, 11(1), 1–16.

[bibr47-08862605251339636] Sandberg D. A. Valdez C. E. Engle J. L. Menghrajani E. (2019). Attachment anxiety as a risk factor for subsequent intimate partner violence victimization: A 6-month prospective study among college women. Journal of Interpersonal Violence, 34(7), 1410–1427. 10.1177/0886260516651314 27226014

[bibr48-08862605251339636] Sesar K. Šimic N. Dodaj A. (2015). Differences in symptoms of depression, anxiety and stress between victims and perpetrators of intimate partner violence. Journal of Sociology and Social Work, 3(2), 63–72. 10.15640/jssw.v3n2a7

[bibr49-08862605251339636] Sheridan-Johnson J. Mumford E. Maitra P. Rothman E. F. (2024). Perceived impact of COVID-19 on cyberabuse, sexual aggression, and intimate partner violence among US young adults. Journal of Interpersonal Violence, 39(15–16), 3483–3507. 38379202 10.1177/08862605241233264

[bibr50-08862605251339636] Smoker M. March E. (2017). Predicting perpetration of intimate partner cyberstalking: Gender and the Dark Tetrad. Computers in Human Behavior, 72, 390–396. 10.1016/j.chb.2017.03.012

[bibr51-08862605251339636] Sparks B. Stephens S. Trendell S. (2023). Image-based sexual abuse: Victim-perpetrator overlap and risk-related correlates of coerced sexting, non-consensual dissemination of intimate images, and cyberflashing. Computers in Human Behavior, 148, 107879. 10.1016/j.chb.2023.107879

[bibr52-08862605251339636] Szabo M. Lovibond P. F. (2022). Development and psychometric properties of the DASS-Youth (DASS-Y): An extension of the depression anxiety stress scales (DASS) to adolescents and children. Frontiers in Psychology, 13, 766890. 10.3389/fpsyg.2022.766890 35496218 PMC9047499

[bibr53-08862605251339636] Tarriño-Concejero L. García-Carpintero-Muñoz M. Barrientos-Trigo S. Gil-García E. (2023). Dating violence and its relationship with anxiety, depression, and stress in young Andalusian University students. Enfermería Clínica (English Edition), 33(1), 47–59. 10.1016/j.enfcle.2022.07.004 36049645

[bibr54-08862605251339636] Tein J. Y. Coxe S. Cham H. (2013). Statistical power to detect the correct number of classes in latent proﬁle analysis. Structural Equation Modeling, 20(4), 640–657. 10.1080/10705511.2013.824781 24489457 PMC3904803

[bibr55-08862605251339636] Terrazas-Carrillo E. Sabina C. (2019). Dating violence attitudes among Latino college students: An examination of gender, *machismo*, and *marianismo*. Violence and Victims, 34(1), 194–210. 10.1891/0886-6708.vv-d-17-00172 30808801

[bibr56-08862605251339636] Terrazas-Carrillo E. Sabina C. Vásquez D. A. Garcia E. (2023). Cultural correlates of dating violence in a combined gender group of Latino College Students. Journal of Interpersonal Violence, 39(3–4), 785–810. 10.1177/08862605231198241 37815051

[bibr57-08862605251339636] Tharshini N. K. Ibrahim F. Kamaluddin M. R. Rathakrishnan B. Che Mohd Nasir N. (2021). The link between individual personality traits and criminality: A systematic review. International Journal of Environmental Research and Public Health, 18(16), 8663. 34444412 10.3390/ijerph18168663 PMC8391956

[bibr58-08862605251339636] Thompson R. S. Bonomi A. E. Anderson M. Reid R. J. Dimer J. A. Carrell D. Rivara F. P. (2006). Intimate partner violence: Prevalence, types, and chronicity in adult women. American Journal of Preventive Medicine, 30(6), 447–457. 16704937 10.1016/j.amepre.2006.01.016

[bibr59-08862605251339636] Toplu-Demirtaş E. May R. W. Seibert G. S. Fincham F. D. (2020). Does cyber dating abuse victimization increase depressive symptoms or vice versa? Journal of Interpersonal Violence, 37(11–12), NP9667–NP9683. 10.1177/0886260520984261 33377407

[bibr60-08862605251339636] Trub L. Starks T. J. (2017). Insecure attachments: Attachment, emotional regulation, sexting and condomless sex among women in relationships. Computers in Human Behavior, 71, 140–147. 10.1016/j.chb.2017.01.052

[bibr61-08862605251339636] Turel O. Qahri-Saremi H. (2018). Explaining unplanned online media behaviors: Dual system theory models of impulsive use and swearing on social networking sites. New Media & Society, 20(8), 3050–3067.

[bibr62-08862605251339636] Ulloa E. C. Martinez-Arango N. Hokoda A. (2014). Attachment anxiety, depressive symptoms, and adolescent dating violence perpetration: A longitudinal mediation analysis. Journal of Aggression, Maltreatment & Trauma, 23(6), 652–669. 10.1080/10926771.2014.920452

[bibr63-08862605251339636] Van Ouytsel J. Ponnet K. Walrave M . (2018). Cyber dating abuse victimization among secondary school students from a lifestyle-routine activities theory perspective. Journal of interpersonal violence, 33(17), 2767-2776. 26872506 10.1177/0886260516629390

[bibr64-08862605251339636] Villagrán A. M. Santirso F. A. Lila M. Gracia E. (2024). Attitudes toward intimate partner violence against women in Latin America: A systematic review. Trauma, Violence, & Abuse, 25(3), 2065–2077. 10.1177/15248380231205825 37897366

[bibr65-08862605251339636] Víllora B. Navarro R. Yubero S. (2021). The role of social-interpersonal and cognitive-individual factors in cyber dating victimization and perpetration: Comparing the direct, control, and combined forms of abuse. Journal of Interpersonal Violence, 36(17–18), 8559–8584. 10.1177/0886260519851172 31140341

[bibr66-08862605251339636] Wadgave U. Khairnar M. R. (2019). Parametric test for non-normally distributed continuous data: For and against. Electronic Physician, 11(2), 7468–7470. 10.19082/7468

[bibr67-08862605251339636] Walsh Z. Swogger M. T. Walsh T. Kosson D. S. (2007). Psychopathy and violence: Increasing specificity. Netherlands Journal of Psychology, 63(4), 125–132. 10.1007/bf03061075 20148183 PMC2817979

[bibr68-08862605251339636] Wissing B. G. Reinhard M. A. (2017). The Dark Triad and the PID-5 Maladaptive personality traits: Accuracy, confidence and response bias in judgments of veracity. Frontiers in Psychology, 8, 1549. 10.3389/fpsyg.2017.01549 28983264 PMC5613765

[bibr69-08862605251339636] Yu R. Pepler D. J. van de Bongardt D. Josephson W. L. Connolly J. (2018). Internalizing symptoms and dating violence perpetration in adolescence. Journal of Adolescence, 69, 88–91. 30278320 10.1016/j.adolescence.2018.09.008

[bibr70-08862605251339636] Zweig J. M. Lachman P. Yahner J. Dank M. (2013). Correlates of cyber dating abuse among teens. Journal of Youth and Adolescence, 43, 1306–1321. 10.1007/s10964-013-0047-x 24198083

